# Rapid movements in plants

**DOI:** 10.1007/s10265-020-01243-7

**Published:** 2021-01-07

**Authors:** Hiroaki Mano, Mitsuyasu Hasebe

**Affiliations:** 1 Division of Evolutionary Biology, National Institute for Basic Biology, Nishigonaka 38, Myodaiji, Okazaki, Aichi 444-8585 Japan; 2 School of Life Science, Graduate University for Advanced Studies, Nishigonaka 38, Myodaiji, Okazaki, Aichi 444-8585 Japan; 3 JST, PRESTO, Honcho 4-1-8, Kawaguchi, Saitama 332-0012 Japan

**Keywords:** Electrical signal, Ion transport, Mechanosensing, Rapid movement, Structure, Water transport

## Abstract

Plant movements are generally slow, but some plant species have evolved the ability to move very rapidly at speeds comparable to those of animals. Whereas movement in animals relies on the contraction machinery of muscles, many plant movements use turgor pressure as the primary driving force together with secondarily generated elastic forces. The movement of stomata is the best-characterized model system for studying turgor-driven movement, and many gene products responsible for this movement, especially those related to ion transport, have been identified. Similar gene products were recently shown to function in the daily sleep movements of pulvini, the motor organs for macroscopic leaf movements. However, it is difficult to explain the mechanisms behind rapid multicellular movements as a simple extension of the mechanisms used for unicellular or slow movements. For example, water transport through plant tissues imposes a limit on the speed of plant movements, which becomes more severe as the size of the moving part increases. Rapidly moving traps in carnivorous plants overcome this limitation with the aid of the mechanical behaviors of their three-dimensional structures. In addition to a mechanism for rapid deformation, rapid multicellular movements also require a molecular system for rapid cell-cell communication, along with a mechanosensing system that initiates the response. Electrical activities similar to animal action potentials are found in many plant species, representing promising candidates for the rapid cell–cell signaling behind rapid movements, but the molecular entities of these electrical signals remain obscure. Here we review the current understanding of rapid plant movements with the aim of encouraging further biological studies into this fascinating, challenging topic.

## Introduction

Both animals and plants exhibit a variety of macroscopic movements either autonomously or in response to external stimuli. In animals, the movement of body parts is indispensable for most vital activities, which range from the involuntary heartbeat to locomotion and foraging behaviors. Movements in plants, such as the growth of shoots towards the sunlight, are usually much slower than those in animals to such a degree that it is difficult to notice them without time-lapse observation. Several plant species, however, have acquired the ability to move as rapidly as animals. In this review, we summarize the current knowledge on rapid plant movements, especially focusing on those actuated by biologically-active hydraulic processes, from a biomechanical and molecular biological perspective.

## Principles of plant movements

**Fig. 1:**
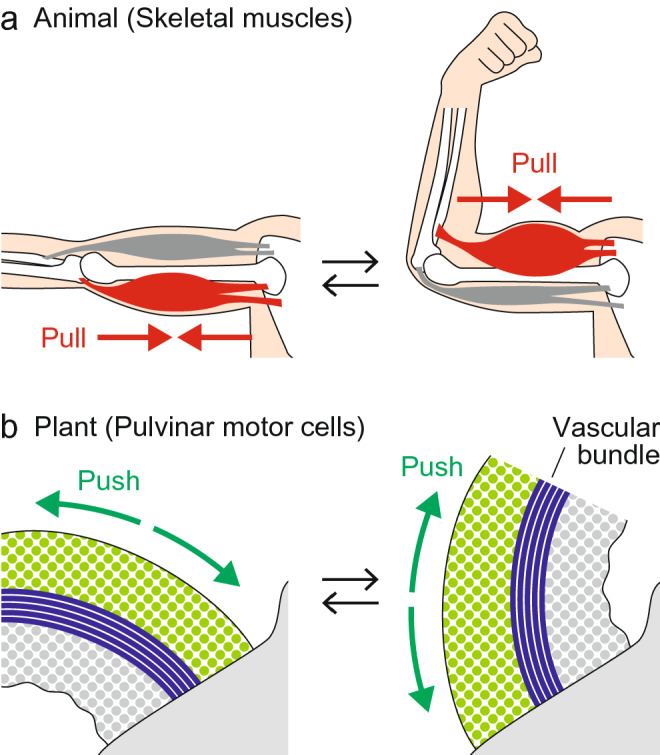
Comparison of animal and plant movements. In animals, contractile forces generated by muscles, together with bones and joints, drive various movements such as bending, stretching, and twisting (**a**). By contrast, plant movements primarily rely on the gain and/or loss of pushing forces exerted by cells and tissues (**b**). In both systems, the antagonistic actions of two opposite sides commonly facilitate movement

Muscular movements in animals rely on the contraction of myofibrils, which is driven by the sliding of actin and myosin filaments alongside each other. Muscles only generate mechanical forces needed to pull and never to push something away. Thus, when we bend our arms, muscles on one side of the arm contract while those on the other side relax (Fig. 1a). When the arm is stretched, these muscles act in an opposite manner. The antagonistic actions of muscles, together with skeletal and joint elements, give animals the ability to move rapidly, reversibly, and repeatedly.

In contrast to the muscular system in animals, the primary force driving plant movements is hydrostatic pressure, i.e., a force that pushes (Fig. 1b). Plants lack a contractile apparatus like myofibrils. Instead, the cell wall surrounding each cell provides an alternative way to generate mechanical forces. The presence of cell walls allows plant cells to maintain higher hydrostatic pressure than animal cells and to strongly alter the pressure without the risk of the cell bursting (Dumais and Forterre 2011; Niklas and Spatz 2012; Taiz et al. 2015).

**Fig. 2:**
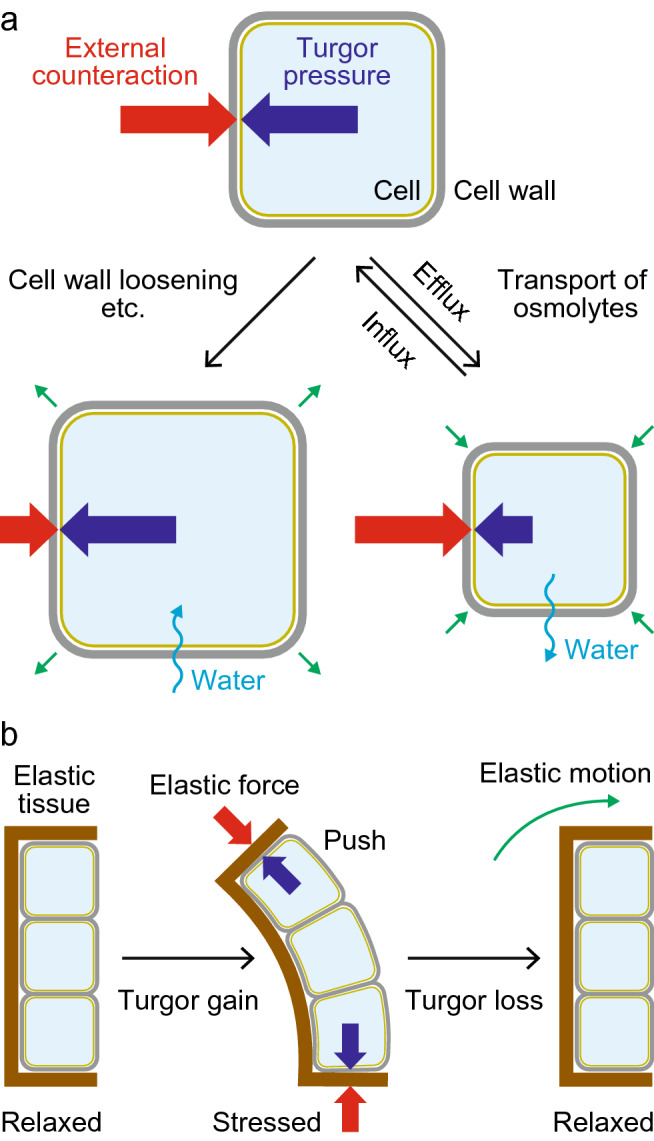
Principles of plant movement. **a** Swelling or shrinking of a cell driven by the loss of balanced forces. Plant cells can maintain their integrity under a wide range of pressure differences due to the presence of the cell wall. **b** The cooperative action of turgor changes and elastic behaviors. Deformation of elastic tissues produces an elastic restoring force, leading to an elastic motion when the balanced force diminishes. The same mechanism underlies the shrinking of a single cell (**a**), in which the elastic restoring force of the expanded cell wall produces external compressive force

The turgor pressure in a cell is generated by the difference in osmotic content between the inside and outside of the cell. At equilibrium, the turgor pressure is balanced by external counteraction (Fig. 2a). A variety of factors contribute to this external counteraction, including the elastic restoring force of the cell wall, mechanical interactions between neighboring cells, and the force of gravity. Movement occurs when the balance of these forces is altered, which can occur via two different mechanisms. One mechanism involves a change in external forces as represented by the loosening of the cell wall (Fig. 2a) (Dumais and Forterre 2011; Forterre 2013). This process leads to the irreversible expansion of the cell and forms the common basis for many types of growth movements (Taiz et al. 2015). The other mechanism involves a change in turgor pressure due to changes in the concentrations of intracellular osmolytes (Fig. 2a) (Dumais and Forterre 2011; Forterre 2013). Movements that depend solely on changes in turgor pressure occur reversibly and repeatedly many times in a plant’s lifetime. The opening and closing of stomata (Jezek and Blatt 2017) and daily leaf movements in legumes (Satter and Galston 1981) are well-known examples of turgor-driven plant movements.

In addition to these direct forces, plants use various types of elastic forces in an indirect fashion to drive movement. Plant cell walls contain elastic materials that enable plant tissues to behave elastically (Treitel 1946); cell walls become stretched, bent, or distorted in a more complicated manner when external or internal forces, such as increased turgor pressure, are applied. These deformed tissues store elastic energy and can passively be restored to their original state when the forces are removed (Fig. 2b). When large amounts of elastic energy that were stored during a long-term build-up phase are suddenly released, some plants execute those movement over a short period (Forterre et al. 2005; Vincent et al. 2011b; Westermeier et al. 2018). It is not surprising that these types of “prepared” movements occur only in previously determined directions and also show all-or-none responses. This feature may represent another difference between rapid plant movements and those performed by animal muscular systems, which can satisfy the speed and flexible control of the movements at the same time.

## Stomatal movement: a model system for plant movement

Stomata are micrometer-size shutters that control the balance between CO_2_ uptake for photosynthesis and water loss due to transpiration (Taiz et al. 2015). To adapt to ever-changing environmental conditions, stomata must open and close rapidly and appropriately in response to external and internal cues. Stomatal opening and closure are completed on a time scale of minutes to hours (Drake et al. 2013; McAusland et al. 2016). Because of their relatively rapid kinetics and their wide distribution among land plants, stomata have become the best-studied model system for investigating both the physiological and molecular mechanisms of plant movements (Jezek and Blatt 2017; Kollist et al. 2014; Lawson and Vialet-Chabrand 2019).

**Fig. 3:**
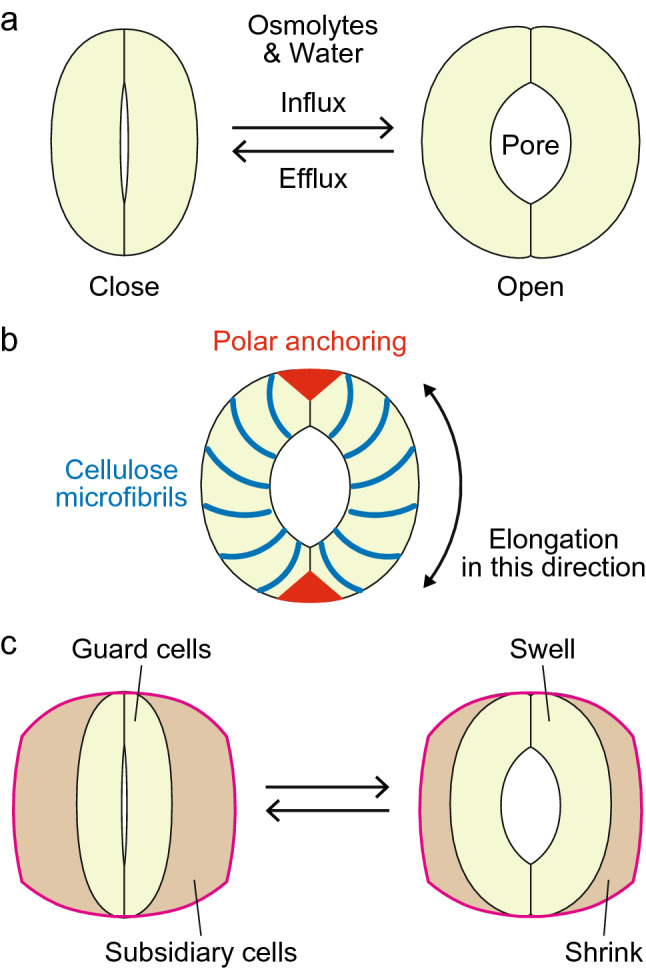
Stomatal movements. **a** Opening and closing of the stomatal pore via the turgor-induced movement of guard cells. **b** Structural elements linking changes in the volume of guard cells to their bending movements. Circumferentially oriented cellulose microfibrils prevent the guard cells from radially expanding. Polar anchoring at both ends prevents their straight expansion. **c** Stomatal complex with subsidiary cells. In this system, swelling and shrinking of the guard cells are accompanied by the reciprocal changes in volume of the subsidiary cells. Note that the outer boundary of the stomatal complex is less altered during these movements

Stomatal movements are mediated by the deformation of guard cells, a specialized pair of cells surrounding each stomatal pore (Fig. 3a). Osmotically driven water uptake increases the volume of guard cells, making them bend and leading to pore opening, whereas the release of osmolytes and subsequent water efflux from guard cells cause the stomata to close (Fig. 3a). K^+^ ions and their counter-anions such as Cl^−^, NO^3−^, and malate are the major osmolytes that regulate the turgor pressure in guard cells. The relative contributions of these ions and non-ionic osmolytes such as sucrose change drastically depending on the time of the day and environmental conditions (Talbott and Zeiger 1996).

**Fig. 4:**
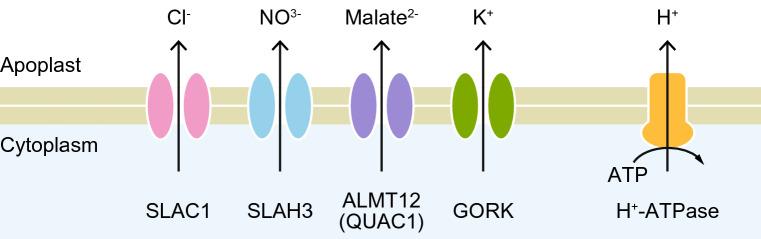
Ion channels responsible for stomatal closure in *A. thaliana*. Representative ions that permeate the channels are also indicated. Electrochemical gradients of ions before stomatal closure are energized by the proton gradient produced by H^+^-ATPases in combination with the activities of various transporters

In the past decade, many ion channels, transporters, and their regulatory components involved in stomatal movement have been identified in the model plant *Arabidopsis thaliana* (Fig. 4). The S-type anion channel SLAC1 is a major contributor to rapid stomatal closure (Negi et al. 2008; Vahisalu et al. 2008). In response to abscisic acid (ABA), which mediates drought stress signaling, SLAC1 causes the efflux of intracellular anions, preferentially Cl^−^ ions, in vivo (Geiger et al. 2009, 2010, 2011). SLAH3, a channel closely related to SLAC1, also contributes to the ABA-stimulated efflux of NO^3−^ ions (Geiger et al. 2011). Malate efflux is mediated by the R-type anion channel ALMT12 (QUAC1), which is also regulated by ABA signaling (Meyer et al. 2010; Sasaki et al. 2010). The efflux of these anions leads to membrane depolarization, which in turn activates voltage-gated K^+^ efflux channels such as GORK (Hosy et al. 2003). Consequently, the guard cells discharge intracellular water, thereby reducing the volume of guard cells, resulting in the closure of the stomatal pore (Fig. 3a). In contrast to stomatal closure mediated by passive ion efflux, stomatal opening requires active ion transport into the cells against the concentration and/or membrane potential gradients. These active processes are energized by plasma membrane H^+^-ATPases (Yamauchi et al. 2016) coupled with various types of ion channels and transporters (Jezek and Blatt 2017). For more detailed information about the effects of ion transport on guard cells, please see (Hedrich 2012; Jezek and Blatt 2017; Saito and Uozumi 2019).

In concert with the molecular systems that control turgor pressure, the mechanical structures of the guard cell play pivotal roles in stomatal movement. Circumferentially oriented cellulose microfibrils prevent the guard cell from expanding in a radial direction (Aylor et al. 1973; Galatis and Apostolakos 2004) (Fig. 3b). Consequently, the guard cell preferentially elongates along the longitudinal axis as the cellular volume increases. In addition to this mechanical feature, the ends of guard cells are anchored in place, which is likely reinforced by the site-specific stiffening of the cell walls in the polar regions of the cells (Carter et al. 2017) (Fig. 3b). The anchoring at both ends of the guard cell prevents the straight-line expansion of this cell, thereby promoting the bending movement to enhance stomatal movement in response to increases in guard cell volume (Aylor et al. 1973; Woolfenden et al. 2018). Asymmetric thickening of the cell wall at the pore side of the guard cell had long been considered to promote this bending movement. However, a recent study involving mathematical modeling and the direct measurement of cell wall stiffness by atomic force microscopy disproved this theory (Carter et al. 2017). Instead, the asymmetric thickening might help relieve mechanical damage caused by the repeated opening and closing of the stomata, as the pore-side cell wall is exposed to large amounts of stress during stomatal opening (Carter et al. 2017).

In some plant species, subsidiary cells surrounding guard cells further enhance the movement of stomata. For example, grasses contain dumbbell-type stomata that open and close due to reciprocal changes in volume between guard cells and subsidiary cells during their movement (Franks and Farquhar 2007): the guard cells expand and the subsidiary cells shrink during stomatal opening, whereas the opposite process occurs during stomatal closure (Fig. 3c). A possible advantage of this system is that the lateral displacement of guard cells is less hindered by the presence of immovable epidermal cells compared to stomata surrounded by guard cells and no subsidiary cells. The functional importance of subsidiary cells was confirmed by a recent genetic study using the wheat relative *Brachypodium distachyon* (Raissig et al. 2017). Mutations in *BdMUTE*, encoding a basic helix-loop-helix transcription factor, led to the lack of subsidiary cells. The mutant stomata exhibited reduced aperture, reduced conductance, and slower responses to changing environmental conditions compared to the wild type (Raissig et al. 2017). The molecular mechanism underlying the rapid seesaw movement between guard cells and subsidiary cells remains to be elucidated. The short-distance shuttle transport of osmolytes between these cells might contribute to this movement (Chen et al. 2017; Franks and Farquhar 2007).

## Pulvini: a representative motor organ composed of many cells

The basal part of a leaf or its derivatives is sometimes modified into a hinge-like structure. In several plant lineages, a self-movable, thickened structure forms at this position called the pulvinus. In many leguminous species, pulvini participate in daily leaf movements in which the compound leaf folds its leaflets at the beginning of the night and unfolds them in the morning (Mayer et al. 1985; Palmer and Asprey 1958; Satter and Galston 1981). This type of movement is known as nyctinastic movement (Minorsky 2019), or more simply sleep movement in reference to human sleep behavior. In addition to sleep movement, the sensitive plant (*Mimosa pudica*) and its close relatives exhibit seismonastic movement (Fleurat-Lessard 1988; Hagihara and Toyota 2020; Roblin 1979), which is completed within seconds after receiving a stimulus (Volkov et al. 2010). Another example of rapid pulvinar movements is found in the telegraph plant (*Codariocalyx motorius*), which continuously rotates its small lateral leaflets at a speed easily observed by our eyes (Darwin 1880); this movement is also triggered by external sounds (Miwa and Yamashita 2001).

**Fig. 5:**
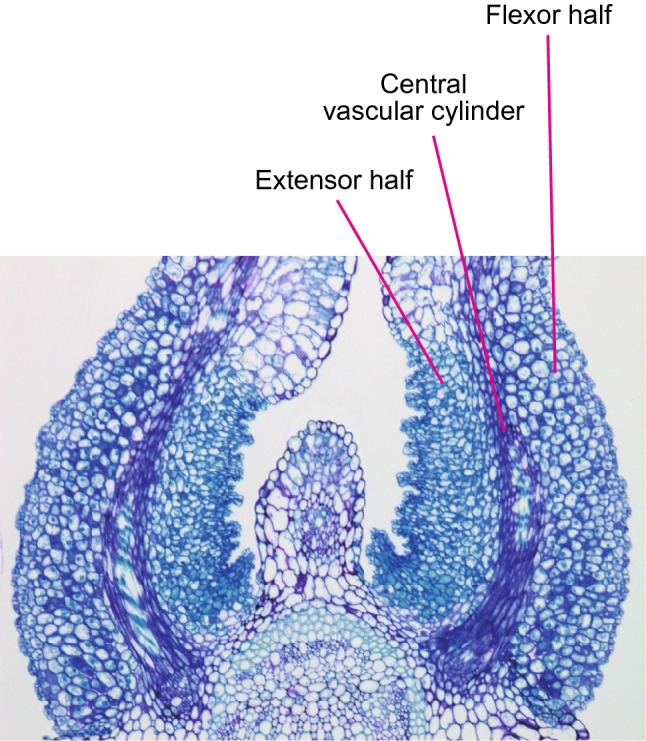
Transverse section of tertiary pulvini of *Mimosa pudica*. The section was stained with toluidine blue. Difference in staining properties between extensor (pale blue) and flexor motor cells (purplish blue) suggest that their cell-wall compositions differ

The movements of pulvini and stomata have similar characteristics, such as being turgor-driven, coupled with bending deformation, and repeatable many times. However, an obvious difference exists in their cellular organization: Guard cells bend and stretch largely in a cell-autonomous fashion, whereas the multicellular movement of a pulvinus involves thousands of cells (Fleurat-Lessard 1988) (Fig. 5). Each pulvinus contains a centralized vascular cylinder, an outermost epidermal cell layer, and parenchymal cell layers between these structures. These parenchymal cells undergo changes in turgor pressure and volume that eventually cause the pulvinus to bend, and hence they are known as pulvinar motor cells. The functional asymmetry of the motor cells in a pulvinus plays a pivotal role in bending movement: motor cells in one half of the pulvinus shrink while those in the other half swell. The halves that swell and shrink during leaf unfolding are traditionally referred to as the extensor and flexor halves, respectively (Satter and Galston 1981) (Fig. 5). Therefore, extensor cells shrink and flexor cells swell during leaf folding.

Despite the multicellular nature of pulvini, the mechanical elements that facilitate their movement can be extrapolated from the mechanics of stomatal movement. Like circumferentially oriented cellulose microfibrils in guard cells, tensile stiffness in the epidermis limits the radial expansion of the pulvinus, whereas the expansion along the longitudinal axis can partially be attributed to the presence of wrinkles on the surface (Fig. 5) (Song et al. 2015). The central vascular cylinder is considered to be functionally equivalent to the polar anchoring of guard cells, as its lower stretchability compared to other cells and its longitudinal linear alignment limit the straight-line expansion of the pulvinus. Consequently, the expansion or shrinkage of one side of the pulvinus causes bending movement, which forces the opposite change on the other side (Fig. 1b) (Asprey and Palmer 1955). This mechanical interaction between the two opposite sides of the pulvinus allows these sides to function antagonistically, in a manner analogous to the antagonistic action of skeletal muscles (Fig. 1a).

A recent study using *Samanea saman* revealed the similarity of nyctinastic movement to stomatal movement at the molecular level (Oikawa et al. 2018). The S-type anion channel gene *SsSLAH1* is predominantly expressed in flexor cells (in the abaxial half of the tertiary pulvinus) of *S. saman*, where its transcripts show a circadian oscillation with a peak in the morning (Oikawa et al. 2018). SsSLAH1 constitutes a functional Cl^−^ channel only when it is present with another S-type anion channel protein, SsSLAH3 (Oikawa et al. 2018). Similarly, the heteromerization of SLAH1 and SLAH3 facilitates Cl^−^ efflux from root pericycle cells in *A. thaliana* (Cubero-Font et al. 2016). In addition, the expression of the *GORK*-like K^+^ channel gene *SPORK2* is enriched in the tertiary pulvinus of *S. saman*, where it displays a circadian oscillation similar to that of *SsSLAH1* (Oikawa et al. 2018). These observations indicate that the asymmetric, clock-controlled expression of ion channel genes regulates nyctinastic movements, possibly in conjunction with the post-translational regulation of channel activities, as observed in stomata.

## **Rapid leaf movement in*Mimosa pudica***: **close to the upper limit of water transport**

As described above, the transport of osmolytes across the plasma membrane and the subsequent movement of water driven by osmosis can account for slow turgor-driven movements that are completed in minutes to hours. One might think that any of the known rapid movements can also be accomplished by simply scaling up the transport systems, for example by increasing the activity and/or amounts of ion channels. However, this is not as simple as it seems, especially for multicellular movements that are completed within seconds or less (Forterre 2013). When multicellular tissues swell or shrink, water moves not only into or out of individual cells but also between tissues at a distant location. The long-distance transport of water through plant tissues imposes a speed limitation on purely hydraulic movements in plants. The maximum speeds of purely hydraulic movements were estimated based on a mathematical analysis of water movement through a porous elastic material (Skotheim and Mahadevan 2005). The calculated timescale of the fastest movement is proportional to the square of the length of the moving part, indicating that the time required for hydraulic movement increases with tissue size (Skotheim and Mahadevan 2005). This calculation also separates naturally occurring movements in plants into two categories: those achievable using hydraulic movements alone and those relying on some type of structural instability to go beyond the hydraulic boundary (Dumais and Forterre 2011; Skotheim and Mahadevan 2005).

The sensitive plant (*M. pudica*) folds its leaves within a few seconds in response to touch or other external stimuli. This seismonastic movement is considered to represent a defense behavior against threats from animals such as herbivores (Braam 2004) and occurs hundreds of times more rapidly than the sleep movements commonly observed in legumes. The speed of the seismonastic movement is close to the upper limit for hydraulic movements (Dumais and Forterre 2011; Skotheim and Mahadevan 2005). As observed for sleep movements, the efflux of K^+^ and Cl^−^ ions from extensor motor cells (Kumon and Suda 1984) and the translocation of water from the extensor half to the flexor half of the pulvinus during seismonastic movement (Tamiya et al. 1988) have been reported. These observations suggest that the hydraulic mechanism dominates seismonastic movement, but whether the movement is a purely hydraulic one (Kwan et al. 2013) or is reinforced by some yet-to-be characterized mechanism (Forterre 2013) is a matter of debate. When we attempt to explain the change in volume of the motor cells based solely on ion efflux across the plasma membrane and the subsequent osmotic flow, the theoretical value of the ion flux becomes at least an order of magnitude higher than the values measured in plant cells or estimated for plant ion channels with the highest conductance (Forterre 2013). One possible explanation for this gap is that perhaps *M. pudica* has achieved an extremely high rate of ion conductance by increasing the performance and/or amounts of ion channels. Alternatively, this plant might recruit another mechanism that does not require the movement of osmolytes, such as the use of contractile proteins (Morillon et al. 2001), active water transport against the osmotic gradient by solute-water cotransporters (Morillon et al. 2001), or rapid loosening of the cell wall (Forterre 2013). Another possibility is that cell-to-cell ion transport occurs in *M. pudica* via symplastic bulk flow through plasmodesmata.

Again, the extensor and flexor halves of a pulvinus mechanically interact with each other due to structural constraints, especially that imposed by the central vascular cylinder. Under resting conditions, when both halves are turgid, they push against one another, and hence the pushing force produced by one half acts as an external compressive force on the opposite half (Asprey and Palmer 1955). When a stimulus is received, a decrease in turgor pressure assumes to be triggered in the extensor motor cells, likely via ion efflux through the activated ion channels. This decrease in turgor pressure in the extensor half should simultaneously cause the compressive force on the flexor half to decrease through a mechanical interaction, which in turn allows the flexor motor cells to expand by absorbing water, without the need for osmolyte transport. During these processes, if the extensor and flexor cells constitute a cytoplasmic continuum with plasmodesmata, the flexor cells potentially draw up water from the extensor cells through the symplastic pathway. This pressure-driven bulk flow through the plasmodesmata may transport small osmolytes together with water, and hence accelerate the shrinking of the cells without being restricted by the ion transport capacity across the membrane. Indeed, ultrastructural studies demonstrated that both the flexor and extensor motor cells of *M. pudica* pulvini possess plasmodesmata (Fleurat-Lessard and Millet 1984), pointing to a possible role for this cytoplasmic connection in movement. However, it remains unclear whether the extensor and flexor cells are connected to each other, and the amounts of water and osmolytes that can actually move through the plasmodesmata are currently unknown.

As well as the hydraulic mechanism, our knowledge of the molecular dynamics of seismonastic movement is currently limited. One of the best-characterized phenomena is the rapid fragmentation of the actin cytoskeleton in extensor motor cells, which is associated with the dephosphorylation of its tyrosine residues (Kameyama et al. 2000; Kanzawa et al. 2006). Treatment with actin-modulating drugs affects the bending angle of seismonastic movement (Kanzawa et al. 2006), indicating that this cytoskeletal element is involved in these movements. An interesting scenario is that actin dynamics might directly contribute to the rapidness of seismonastic movement, for example by causing a sudden change in mechanical forces in motor cells, but there is currently no experimental support for this idea. Instead, actin dynamics appear to have a role in the mechanism for electrically stimulated Ca^2+^ influx into motor cells, a key event that triggers their contraction (Yao et al. 2008). A similar interaction between actin dynamics and Ca^2+^ influx was observed on the plasma membranes of stomatal guard cells (Zhang et al. 2007), suggesting that actin dynamics play a part in a molecular event that occurs on the membrane.

## Movements of carnivorous plants: beyond the upper limit of water transport

Seismonastic movements in *M. pudica* are unusually dynamic and rapid compared to other plant movements, but they can still be observed by the human eye. Several plant species exhibit much more rapid movements, at subsecond to millisecond time scales, the details of which can only be observed through slow-motion replay. A representative example of such ultrafast movements is the movement of trap leaves in carnivorous plants, which must complete their capture motion before the animal prey escapes. Studies of these movements have revealed how plants overcome the speed limitation imposed by water transport and how the 3D structures of moving parts play essential roles in plant movements.

**Fig. 6:**
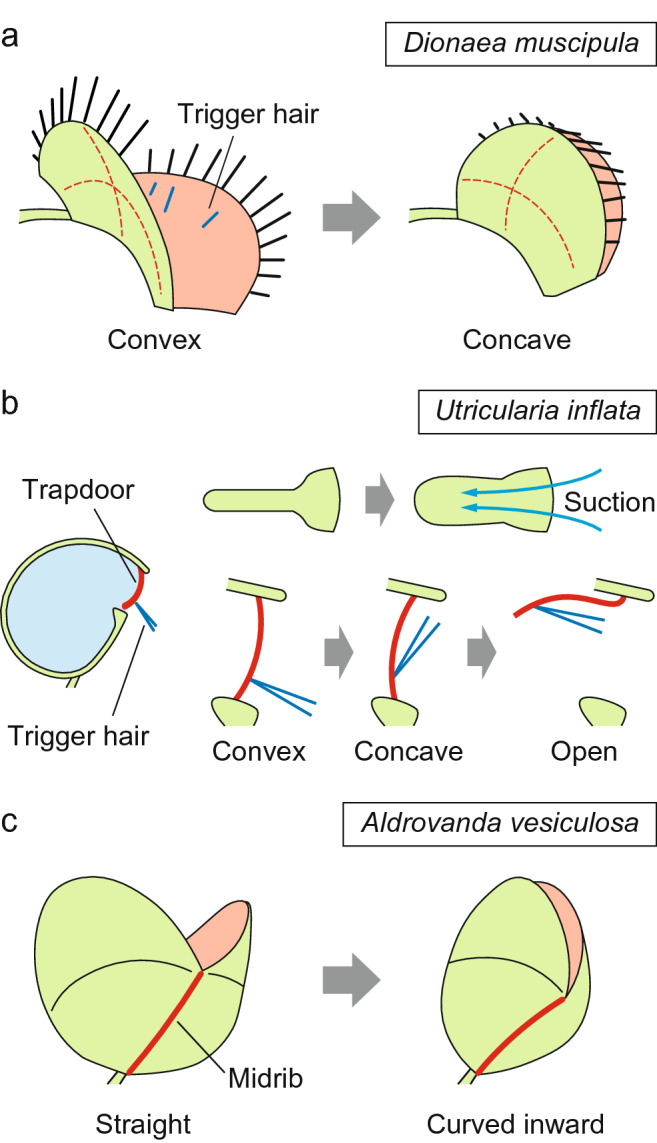
The 3D structures of carnivorous plants contribute to their rapid movements. **a** Snap trap of the Venus flytrap plant. Structural constraints on the trap lobes impose an energy barrier between the convex and concave configurations. Once the stored energy reaches a critical point due to physiological changes, rapid inversion of the curvature occurs. **b** Suction trap of a bladderwort. In the “ready-to-catch” state, negative pressure is maintained inside the trap, leading to the rapid suctioning of water when the trapdoor opens. The trapdoor in convex configuration can resist this negative pressure, but a small mechanical perturbation transmitted by trigger hairs easily changes the curvature of the trapdoor. The trapdoor in the concave configuration is no longer able to seal the trap entrance. **c** Snap trap of a waterwheel plant. Motor cells around the midrib bend it slightly inwards. This small displacement leads to larger angular movements of the trap lobes via the mechanical interlocking between the midrib and lobes

Venus flytrap (*Dionaea muscipula*) has snap-type trap leaves containing a pair of terminal lobes (Fig. 6a). When the sensory trigger hairs on the inner surface of a trap lobe are mechanically stimulated, an electrical signal (an action potential, see the next section) is propagated in the lobes (Hodick and Sievers 1988). The trap does not respond to a single occasional stimulus, perhaps to avoid unwanted triggering by abiotic factors. When the lobes receive two or more successive stimuli within approximately 30 seconds, they suddenly snap shut and trap the prey inside (Brown and Sharp 1910). A recent study employing the fine displacement of the sensory hair showed that a single deflection with relatively slow angular velocity (0.03–4 radian per second) can induce multiple action potentials and thus trigger the trap closure (Burri et al. 2020). Live-imaging analysis using transgenic *D. muscipula* has recently demonstrated that cytosolic Ca^2+^ levels in the trap lobes cumulatively increase in response to individual stimuli, suggesting that Ca^2+^ plays a role in this counting system (Suda et al. 2020). Further mechanical stimuli on the trigger hairs, indicating that prey capture has been successful, cause the trap to close more tightly, ultimately leading to the formation of a liquid-tight “green stomach” filled with digestive fluid (Böhm et al. 2016; Escalante-Perez et al. 2011; Hedrich and Neher 2018; Scherzer et al. 2017). Despite the relatively large size of trap leaves (up to a few centimeters), the shutting movement occurs in only ~ 100 ms (Forterre et al. 2005). This speed exceeds that allowed for purely hydraulic movements (Skotheim and Mahadevan 2005). To achieve this rapid speed, the Venus flytrap takes advantage of the curved structure of the trap lobes (Forterre 2013; Forterre et al. 2005). When viewed from the inner surface, the lobes are convexly curved in the open state, whereas their curvature changes to concave after closing (Fig. 6a). Due to the mechanical instability of the intermediate structures, the lobes prefer to maintain either the convex or concave structure. During the active deformation process, the lobes initially try to maintain an overall convex shape; this action leads to the gradual storage of elastic energy. Once the stored energy reaches a critical point, it is rapidly released and causes the lobes to flip very rapidly. This snap-buckling part of the movement primarily reflects the conversion of elastic energy into kinetic energy, and hence it is not constrained by hydraulic limitations. A recent study indicated that successful snaping of the trap lobes also requires the previous accumulation of internal hydrostatic pressure and the interplay among three different tissues: the expansion of outer epidermis, the shrinkage of inner epidermis, and the neutral behavior of the middle layer that may function to increase the leverage interaction between the outer and inner epidermises (Sachse et al. 2020).

Another example of the use of buckling instability is found in bladderworts (*Utricularia* spp.) (Vincent et al. 2011b). Aquatic species of the genus contain bladder-like traps that suck in their animal prey together with the surrounding water (Fig. 6b). This suction mechanism involves the pumping of water out of the inner space, likely through specialized glands (Poppinga et al. 2016), which generates negative pressure within, with elastic energy stored in the deflated trap body. The entrance of the trap is tightly sealed by an excurved trapdoor equipped with trigger hairs on its outer surface (Fig. 6b). When the trigger hair is mechanically stimulated, the curvature of the door is inverted from convex to concave (Vincent et al. 2011b). The resulting concave structure is no longer tolerant of the negative pressure and the trapdoor swings inward. This causes water to suddenly flow into the trap (Müller et al. 2020). Finally, the trapdoor goes back to the original position to prevent the prey from exiting. The entire movement process takes only a few to tens of milliseconds, representing the fastest movement among flowering plants.

Unlike the triggering and buckling mechanisms used by Venus flytrap, it is likely that the mechanisms used by the bladderwort trapdoor are purely mechanical. The direct displacement of the trigger hair generates a mechanical disturbance on the trapdoor sufficient to cross over the critical point required to trigger buckling. Therefore, a “ready-to-catch” trap must maintain internal pressure at a level immediately below the critical point; otherwise, the trap would not respond to stimulation or would simply repeat the suction step irrespective of the presence of prey. In fact, such spontaneous firings in the absence of triggering by prey have been observed (Vincent et al. 2011a). These spontaneous firings exhibit diverse temporal patterns, from firings at relatively constant intervals to more scattered and random ones; this type of variation is observed even in different traps on the same compound leaf. The firing intervals tend to increase gradually over time, and the traps sometimes make a transition from a metronomic to a random pattern or simply stop firing spontaneously. The trap might fine-tune the balance between the internal pressure and the critical point of buckling through aging and/or certain effects that accumulate after repeated movements, such as mechanical fatigue in the trap body, increases in water leakage, or reinforcement of trapdoor stiffness by cellular metabolism. Notably, the spontaneous firings themselves provide an ecological benefit to the plant as a means of catching immotile small organisms, such as algae and pollen grains (Koller-Peroutka et al. 2015). A recent study also indicated that the friction of water becomes problematic when the suction trap is as small as those of bladderworts (Müller et al. 2020). To reduce the negative effects of water friction, bladderworts generate inertia-dominated flows by employing three biomechanical and morphological adaptations: strong and constant suction pressure made by the elasticity of the trap walls, short entrance channel, and the rapid opening kinetics of the trapdoor described above (Müller et al. 2020).

The waterwheel plant (*Aldrovanda vesiculosa*) is an aquatic carnivorous plant that possesses snap-type traps (Fig. 6c). Because of the overall similarity of the snap traps of Venus flytrap and *A. vesiculosa* and the phylogenetic sister relationship of the two species (Cameron et al. 2002; Rivadavia et al. 2003), the snap trap of *A. vesiculosa* is often regarded as just a downsized version of that of Venus flytrap. However, the mechanics of their rapid closure differ substantially (Poppinga and Joyeux 2011; Westermeier et al. 2018). *A. vesiculosa* snap traps do not employ a snap-buckling mechanism in their movements. Instead, the trap lobes are already concave in the open state, and the curvature remains essentially unchanged during movement (Fig. 6c). Although the speed of the movement (20–100 ms) (Westermeier et al. 2018) is comparable to or faster than that of Venus flytrap, it falls within the range of hydraulic movements (Dumais and Forterre 2011; Skotheim and Mahadevan 2005) because of the smaller size of the trap (2–4 mm). Despite of these fundamental differences, the 3D structure of the trap plays a pivotal role in the movement of *A. vesiculosa* as well (Poppinga and Joyeux 2011). In this species, the motor tissues whose turgor pressure changes are located close to the midrib (Iijima and Sibaoka 1983), a hinge-like region between the lobes. During this movement, the midrib bends inward (Fig. 6c). This displacement itself is quite small, but it is sufficient to induce a large angular movement of the trap lobes via a mechanical interlocking mechanism between the midrib and lobes (Poppinga and Joyeux 2011). This mechanism, known as kinematic amplification, is in principle the same as a lever system, which amplifies displacements together with a proportional decrease in force. This represents an efficient way to accelerate the speed of the displacement, even under hydraulic limitations.

## Molecular mechanisms potentially involved in rapid plant movements

In contrast to the remarkable progress in understanding the biomechanical aspects of rapid plant movements, the molecular and genetic bases for these movements remain largely uncharacterized. This is primarily because rapid movements are generally unique to non-model plants whose genes are difficult to functionally analyze in vivo. However, several genes involved in these rapid movements might have been recruited from other pre-existing systems that are commonly found in many plant species. It would therefore be helpful to gain insights from studies of model plant species, especially studies of the rapid responses of these plants to external stimuli. Except for the purely mechanical system in bladderworts, the rapid movements described above consist of three physiological processes: mechanosensing, rapid signal transduction, and rapid deformation. The current understanding of the molecular and biomechanical aspects of rapid deformation is discussed above. Here, we will focus on mechanosensing and rapid signal transduction.

Mechanical stimulus acts as the major trigger for rapid movements in various plant species, including *M. pudica* and carnivorous plants. Many other plants, although they do not show a visible acute response to touch, do sense and respond to it in less obvious ways (Braam 2004; Chehab et al. 2008). For example, Ca^2+^-imaging analysis of *A. thaliana* revealed that touch or mechanical bending induces an influx of Ca^2+^ into the cytoplasm within seconds (Knight et al. 1991). Touch stimulus also induces acute changes in the expression levels of hundreds of genes (Braam and Davis 1990; Lee et al. 2005), which in turn evoke many physiological responses, such as phytohormone production, cell wall modification, and disease resistance (Chehab et al. 2008).

**Fig. 7:**
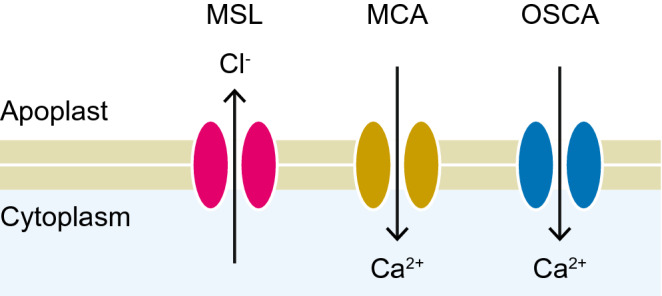
Mechanosensitive ion channel families in plants. Representative ions that permeate the channels are also indicated

Because a mechanical force can influence various aspects of biological systems, there are many possible ways to detect the stimulus at the molecular level (Monshausen and Haswell 2013). Currently, the best-characterized sensors of mechanical forces are mechanosensitive ion channels, which are primarily activated by the stretching of the circumjacent cellular membrane (Hamant and Haswell 2017; Kurusu et al. 2013; Monshausen and Haswell 2013). At least three different families of mechanosensitive channels have been identified in plants (Fig. 7). MSL (MscS-like) family proteins were identified based on their partial homology to bacterial MscS (mechanosensitive channel of small conductance) (Haswell and Meyerowitz 2006) and were subsequently confirmed to constitute stretch-activated ion channels that preferentially permeate anions such as Cl^−^ (Maksaev and Haswell 2012). In *A. thaliana*, MSL2 and MSL3 localize to the plastid envelope and serve to maintain the size and shape of the plastid against hypo-osmotic stress (Haswell and Meyerowitz 2006; Veley et al. 2012). MSL8 localizes to the plasma membranes and endomembranes of pollen grains, where it helps maintain osmotic balance during pollen rehydration and germination (Hamilton et al. 2015). The biological roles of other MSL channels remain elusive due to the lack of identifiable phenotypes associated with them, even in the quintuple mutant *msl4/5/6/9/10*, although an *in vitro* electrophysiological study demonstrated that these MSL proteins are responsible for the mechanosensitive channel activity in root protoplasts (Haswell et al. 2008).

MCA (*mid1*-complementing activity 1) proteins were identified through a functional screen for *A. thaliana* cDNAs that could rescue the lethality of a yeast mutant lacking a mechanosensitive Ca^2+^ channel (Nakagawa et al. 2007). MCA proteins are Ca^2+^-permeable, mechanosensitive channels (Furuichi et al. 2012) unique to the plant kingdom (Kurusu et al. 2013). The roots of the *A. thaliana mca1* null mutant were less able to penetrate a hard agar layer than the wild type, suggesting that MCA1 plays a role in sensing the hardness of soil (Nakagawa et al. 2007). MCA proteins also mediate cold-induced increases in Ca^2+^ levels in the cytosol (Mori et al. 2018). OSCA family proteins were first identified as mechanosensitive channels in plants and were subsequently found to be widely distributed in eukaryotes (Hou et al. 2014; Yuan et al. 2014). OSCAs are stretch-activated (Zhang et al. 2018), non-selective cation channels with no or weak outward rectifying properties (Hou et al. 2014; Murthy et al. 2018; Yuan et al. 2014). *A. thaliana* OSCA1.1 localizes to the plasma membrane and contributes to hyperosmolarity-induced increases in Ca^2+^ levels in the cytosol (Yuan et al. 2014). Loss-of-function mutants of *OSCA1.1* showed impaired stomatal closing and inhibited root growth under high osmolarity conditions (Yuan et al. 2014), suggesting that OSCA1.1 plays a general role in osmosensing. Members of these three families might participate in triggering rapid movements, for example by increasing Ca^2+^ concentrations in the cytosol (MCAs and/or OSCAs), altering the membrane potential (all three families), or directly causing rapid losses in turgor pressure (MSLs).

Rapid cell-to-cell signaling is a key component of rapid movements in multicellular systems. For example, each pulvinus in *M. pudica* contains thousands of motor cells (Fleurat-lessard 1988). Even though individual motor cells can shrink in only 1 s, the entire organ could not move at a similar time scale without a rapid synchronization mechanism for the cells. Some rapid movements are also characterized by long-distance signaling, often through different types of cells. In *M. pudica*, stimulation leads to the propagation of leaf folding, in which unstimulated pulvini sequentially move one after another. In Venus flytrap, two mechanical stimuli on different trigger hairs can cause the trap to close, even if the trigger hairs are located on different lobes in a trap, pointing to the presence of a sophisticated system that integrates multiple inputs from distant places. In animal nervous systems, a neuron rapidly transmits a signal to its neighbors via electrical action potentials. Similar self-propagating, transient changes in membrane potential have been detected in many plant species (Choi et al. 2016; Fromm and Lautner 2007) in addition to classic electrophysiological models such as *M. pudica* (Fromm and Eschrich 1988; Samejima and Sibaoka 1982; Sibaoka 1962), Venus flytrap (Hodick and Sievers 1988), and green algae with giant cells (Lunevsky et al. 1983). Although the propagation speeds of electrical signals in plants (typically ranging from centimeters to millimeters per second) (Choi et al. 2016) are much slower than those of their animal counterparts (up to tens of meters per second), they still represent one of the fastest signaling events in plants. Cells in the vascular system, especially sieve tube elements in the phloem, are thought to be the major pathways of rapid electrical signals (Fromm and Eschrich 1988; Fromm and Lautner 2007; Hedrich et al. 2016; Nguyen et al. 2018; Samejima and Sibaoka 1983). The elongated, cytoplasmically connected cell structures of the vascular system and its network throughout the plant body make the vascular system suitable for long-distance electrical communication, in addition to other systemic signaling mediated by molecular transport or the direct transmission of hydraulic pressure (Choi et al. 2016).

**Fig. 8:**
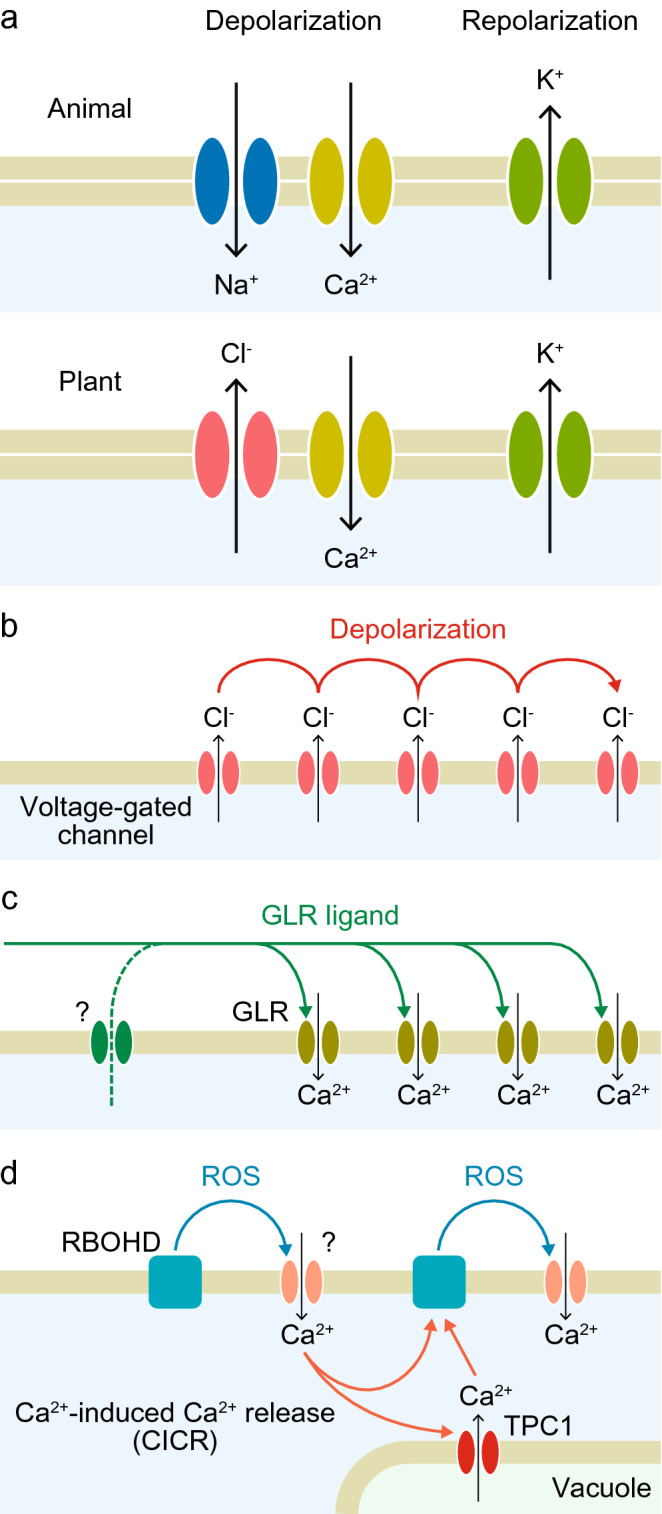
Models for rapid signaling mechanisms in plants. **a** Schematic representation of ion channel activities that generate action potentials in animals and their hypothetical counterparts in plants. **b** Classic action potential model in plants. Depolarization of the plasma membrane activates neighboring voltage-gated channels, leading to further depolarization. **c** Ligand-gated Ca^2+^ influx through GLRs (olive green). GLR ligands come directly from distal sources such as wounded cells or are secreted from cells constituting the signaling pathway through unknown channels or transporters (green). **d** ROS-Ca^2+^ wave. RBOHD is a ROS-producing enzyme whose activity is upregulated by increases in cytoplasmic Ca^2+^ levels. Ca^2+^ influx into the cytoplasm occurs through as-yet-unidentified ROS-activated Ca^2+^ channels on the plasma membrane, and the cytoplasmic Ca^2+^ concentration further increases due to Ca^2+^-induced Ca^2+^ release (CICR) mediated by the vacuolar Ca^2+^ channel TPC1

Action potentials in animal neurons are initiated by the influx of Na^+^ and Ca^2+^, which increases the membrane potential (depolarization) (Fig. 8a). Subsequently, the efflux of K^+^ occurs and the membrane potential returns to the initial state (repolarization). Both the depolarization and repolarization processes are mediated by voltage-gated channels for the respective ions (Bean 2007). The positive feedback of the voltage-gated Na^+^ channels, in which depolarization leads to the activation of the neighboring channels, allows the action potential to propagate on the plasma membrane. Action potentials in plants are thought to be generated by a fundamentally similar mechanism, which differs in that the depolarization process is caused by the efflux of Cl^−^ and/or the influx of Ca^2+^ (Fromm and Lautner 2007; Hedrich et al. 2016; Sibaoka 1991) (Fig. 8a, b). However, the channels that propagate these signals remain largely unknown. One of the biggest challenges in this field is to identify the voltage-gated ion channels responsible for the depolarization-induced depolarization event. Land plants lack animal- and green algae-type voltage-gated Na^+^/Ca^*2+*^ channels (Edel et al. 2017). As for Cl^−^ channels, an *in vitro* analysis demonstrated that ALMT12 (QUAC1) has a rapid voltage-gated property like that of voltage-gated Na^+^ channels (Meyer et al. 2010), but it is not yet known whether this or related channels generate self-propagating action potentials *in vivo*.

In recent years, glutamate-receptor-like (GLR) channels have attracted attention for their roles in the propagation of wounding-induced slow electrical signals (Mousavi et al. 2013), as well as other cell-to-cell communication systems, including the control of stomatal aperture (Kong et al. 2016; Yoshida et al. 2016) and the chemotactic guidance of pollen tubes (Michard et al. 2011) and sperm cells in moss (Ortiz-Ramírez et al. 2017). Plant GLRs, which are homologous to ionotropic glutamate receptors in animals, function as Ca^2+^-permeable cation channels (Davenport 2002; Forde and Roberts 2014). Like their animal homologs, plant GLRs are gated by extracellular small ligands (Forde and Roberts 2014), possibly in concert with other intracellular regulatory mechanisms (Wudick et al. 2018). One of the features unique to plant GLRs is the broad spectrum of their ligand specificity. Various L-amino acids, which are not confined to glutamate, positively or negatively regulate their channel activities, and each GLR family member has a different preference for ligands (Forde and Roberts 2014; Kong et al. 2016; Michard et al. 2011; Qi et al. 2006; Tapken et al. 2013). In addition to the standard proteinogenic amino acids, glutathione (Qi et al. 2006) and D-serine (Michard et al. 2011) also function as ligands for GLRs, pointing to the potential involvement of various metabolites in the ligand-gated systems. The *A. thaliana glr3.3 glr3.6* double mutant showed defects in systemic electrical signaling induced by wounding, especially in leaf-to-leaf transmission of the signal (Mousavi et al. 2013). This electrical signaling is associated with increases in cytoplasmic Ca^2+^ levels along the vasculature, which was also abolished in the *glr3.3 glr3.6* mutant (Nguyen et al. 2018; Toyota et al. 2018). Live-imaging analysis demonstrated that the glutamate concentration initially increases in response to wounding in the apoplast at the wounding site and then spreads to distal areas (Toyota et al. 2018), suggesting that ligand-gated Ca^2+^ influx though the GLRs, at least in part, constitutes long-distance electrical signaling. It is likely that the apoplastic glutamate is released directly from wounded cells, but it is also possible that cells located along the route actively discharge glutamate through transporters or channels (Dennison and Spalding 2000) (Fig. 8c).

Notably, a cytoplasmic Ca^2+^ wave could be propagated without relying on a voltage-gated system. For example, a reactive oxygen species (ROS)-Ca^2+^ relay, whose signals travel at the speed of hundreds of micrometers per second (Choi et al. 2014), is likely generated by the cooperative action of the vacuolar Ca^2+^ channel TPC1, the ROS-producing enzyme RBOHD, and as-yet-uncharacterized ROS-activated Ca^2+^ channels (Choi et al. 2014; Evans et al. 2016; Gilroy et al. 2016) (Fig. 8d). These recent findings suggest that the generation of electrical signals associated with rapid plant movements is potentially mediated by a mechanism that differs from the classic action potential model.

## Concluding remarks

In this review, we have described the biomechanical and molecular aspects of rapid plant movements, especially those discovered in recent studies. We focused on repeatable movements in living plants, as physiological processes are the primary contributors to these movements. It should be noted that other plant movements function via different principles. For example, the desiccation and/or destruction of tissues can induce very rapid elastic movements (Dumais and Forterre 2011; Forterre 2013), such as the cavitation-triggered catapult of the fern sporangium (Noblin et al. 2012) and explosive seed dispersal (Hofhuis et al. 2016). The coiling mechanisms of seed pods (Armon et al. 2011) and tendrils (Gerbode et al. 2012) also provide valuable insights into plant biomechanics: the asymmetric contraction properties between two layers of these plant structures play an essential role in these mechanisms.

The mechanisms underlying rapid plant movements have been extensively studied for over a century, beginning with the pioneering studies of Darwin (1880) and Pfeffer (1900). In the past two decades, advances in measuring technologies and computer science have led to a much greater understanding of the physics and biomechanics of rapid plant movements. However, many unsolved questions remain that will be accessible only with the aid of molecular biological studies. Biological research in this field has long been hampered by the lack of powerful, efficient tools to analyze gene function *in vivo*. Recent advances in transgenic techniques (Lee et al. 2019; Mano et al. 2014; Oropeza-Aburto et al. 2020; Suda et al. 2020) and whole-genome sequencing (Ibarra-Laclette et al. 2013; Palfalvi et al. 2020) of plants showing rapid movements, together with the rapid development of genome editing technology (Yin et al. 2017), will lead to technical breakthroughs in this compelling, challenging field of research.
